# Green Extraction Strategy Using Bio-Based Aqueous Biphasic Systems for Polyphenol Valorization from Grape By-Product

**DOI:** 10.3390/foods13060954

**Published:** 2024-03-21

**Authors:** Aleksandra Dimitrijević, Slađana Marić, Ana Jocić, Danijela Tekić, Jasmina Mušović, Joana S. Amaral

**Affiliations:** 1 Vinča Institute of Nuclear Sciences—National Institute of the Republic of Serbia, University of Belgrade, Mike Petrovica Alasa 12–14, 11000 Belgrade, Serbia; sladjana.maric@vin.bg.ac.rs (S.M.); ana.jocic@vin.bg.ac.rs (A.J.); danijela.tekic@vin.bg.ac.rs (D.T.); jasmina.musovic@vin.bg.ac.rs (J.M.); 2 Centro de Investigação de Montanha (CIMO), Instituto Politécnico de Bragança, Campus de Santa Apolónia, 5300-253 Bragança, Portugal; jamaral@ipb.pt; 3 Laboratório Associado para a Sustentabilidade e Tecnologia em Regiões de Montanha (SusTEC), Instituto Politécnico de Bragança, Campus de Santa Apolónia, 5300-253 Bragança, Portugal

**Keywords:** resveratrol, quercetin, gallic acid, ionic liquids, extraction, aqueous biphasic systems, polyphenols, grape by-products, food waste

## Abstract

Polyphenols are natural compounds with enhanced antioxidant properties. They are present in relatively high concentrations in fruit/vegetable by-products. Therefore, there is a need for the development of efficient and cost-effective methods for the separation and purification of these valuable compounds. Traditional extraction with organic solvents needs to be switched to novel methods that are more efficient, with reduced extraction times and low consumption of organic solvents. Aiming at developing sustainable processes for the separation and purification of phenolic compounds, we used three model compounds, namely resveratrol, quercetin, and gallic acid, to investigate ionic liquid-based aqueous biphasic systems (IL-ABSs) formed by cholinium-based IL in combination with polypropylene glycol with a molecular mass of 400 g/mol (PPG400). The ABS composition in the two-phase region was selected according to a previously determined phase diagram. Extraction studies indicated the preferential partition of resveratrol and quercetin toward the hydrophobic PPG-rich phase that is mainly dominated by its hydrophobic nature and the strong salting-out effect of ILs. On the other hand, due to its considerably hydrophilic nature, gallic acid preferentially migrates toward the IL phase. The achieved results from grape stem extract demonstrated high extraction efficiencies of cholinium dihydrogen phosphate (~99% for resveratrol for the PPG phase and 78% for gallic acid for the IL phase), with considerable selectivity, demonstrating promising outcomes for potential applications.

## 1. Introduction

Food loss and food waste are currently major problems faced by the food supply chain globally. The Food and Agriculture Organization of the United Nations estimates that approximately one-third of food is either lost or wasted [1]. This poses significant challenges from environmental, economic, and social perspectives. Fruit and vegetable processing industries generate high amounts of “waste”, such as pomace, peels, seeds, and stems [2]. Several purposes have been adopted for these by-product materials, including use as soil fertilizers or animal feed or as biomass to produce energy. However, these fruit and vegetable by-products are rich in a wide range of high-added-value bioactive phytochemicals, mainly polyphenolic compounds [3]. Bioactive polyphenols are on the top of the well-known value pyramid of biomass, which makes food by-products very attractive from a circular bioeconomy perspective [4]. Polyphenolic compounds are scavengers of free radicals, which are products that are harmful to aerobic metabolism, leading to oxidative stress in organisms. Multiple studies have shown that polyphenol compounds exhibit diverse biological activities, including antioxidant and anti-inflammatory properties, cardiovascular health benefits, potential anti-cancer effects, antimicrobial activity, metabolic improvements, and neuroprotective effects [5]. Moreover, dozens of studies have been published recently regarding the possible use of polyphenols to treat SARS-CoV-2 based on previous evidence of phenolic activity against different viruses [6]. Therefore, growing knowledge about polyphenols’ health benefits and increased health awareness among consumers promote the use of these bioactive compounds as natural food additives over synthetic agents. According to Allied Market Research, the polyphenol market was valued at USD 1.6 billion in 2020, and it is projected to reach USD 2.7 billion by 2030, registering a compound annual growth rate of 5.2% from 2021 to 2030 [7]. Therefore, fruit and vegetable by-products are a prevailing source of these valuable compounds, making their valorization a core objective of much current research.

**Figure 1:**
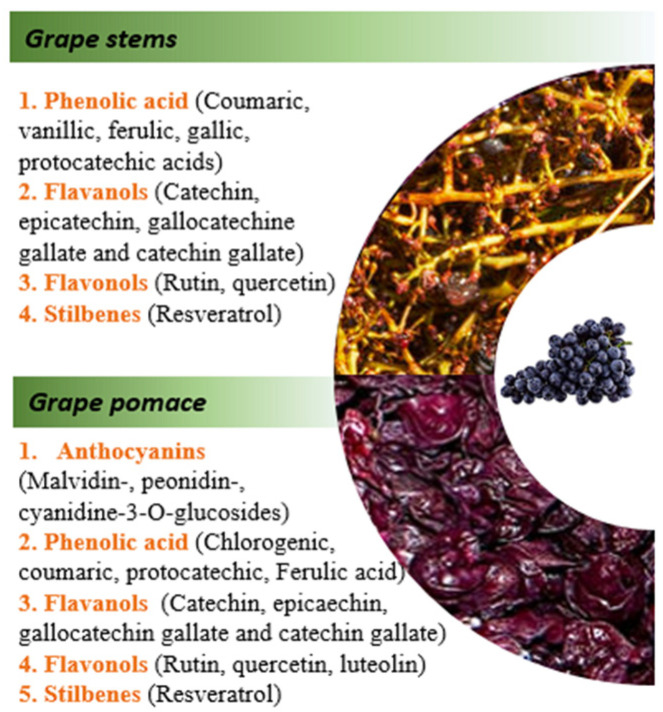
Main phenolic compounds present in selected by-products [11,12].

Grape by-products (grape stems and grape pomace) are among the most investigated agri-food matrices with the most potential due to their exceptional abundance of diverse polyphenols, along with their substantial residue fractions and waste volume [8]. The wine industry is the main contributor of grape by-products, generating 9 million tons of grape waste annually around the world, with the by-products retaining 45–65% of the total polyphenols [9]. Two main by-products obtained from wine cellars are grape pomace, accounting for 10–20% of the processed material, and stems, which constitute about 2–8% [10]. With this consideration, it is rational to view grape by-products as valuable sources of useful chemical compounds rather than mere waste. Polyphenols found in grape by-products include anthocyanins, phenolic acids, flavanols, flavonols, and stilbenes (Figure 1).

Anthocyanins and phenolic acids have a more hydrophilic character, i.e., they are more soluble in water compared to other polyphenolic compounds in by-product matrices. As can be seen from in Figure 1, grape stems are rich in phenolic acid, flavanols, flavonols, and stilbenes [13]. Recent concerns over the safety and adverse health effects of synthetic food additives, particularly on neurological functions and behavior [14], have ignited a strong interest in exploring natural biologically active alternatives. Therefore, by extracting and utilizing these bioactive phytoconstituents, we can reduce the need for synthetic additives or ingredients sourced from non-renewable resources. This promotes a more sustainable and diversified food system, enhancing its capacity to withstand challenges such as resource scarcity and disruptions in the global food supply chain. While grape pomace from wine processing is already industrially utilized to extract anthocyanins, there’s a growing need to further valorize the residues from wine processing. This includes developing methods to extract high-value co-products like natural health remedies, food supplements, and innovative nutrifunctional food ingredients. However, there is still a long way to go until all these residues gain a factual recovery pathway, making the winemaking process a more sustainable activity.

Commercial methods such as solvent extraction (maceration) and Soxhlet extraction are commonly used to extract plant polyphenols. However, these techniques are not environmentally friendly due to their extensive use of organic solvents. Additionally, they are time-consuming and often yield lower extraction efficiencies [15,16]. Alternative methods like ultrasound-assisted extraction and microwave-assisted extraction have gained relevance in recent years, but they necessitate sophisticated equipment that can be challenging to scale up for industrial applications. High-pressure and high-temperature processes are generally avoided, as they can lead to the degradation of thermally unstable bio-active compounds during polyphenol extraction [17,18]. Polyphenols such as gallic acid, resveratrol, and quercetin can undergo significant degradation at higher temperatures, which makes high-temperature methods unsuitable for extraction. For example, degradation of GA begins from 60 °C, with 30% degradation occurring at 100 °C [19,20]. Generally all polyphenolic compounds are prone to pH and temperature-dependent degradation [17,21,22,23]. Moreover, a large fraction of phenolic compounds are left behind after extraction with traditional methods, as they are usually covalently bound to complex polysaccharides in the cell walls of food matrices [24]. In response to these challenges, it is of high importance to develop modern and efficient green solvent technology for extraction, separation, and concentration rooted in the principles of Green Chemistry, focusing on sustainable and environmentally friendly concepts for recovery of high-value polyphenols from selected food by-products. This entails employing efficient and benign solvents, and ideally, developed technology should integrate multiple steps, combining efficiency with sustainability and aligning with current societal and environmental standards.

Ionic liquids (ILs) are molten salts consisting entirely of ions (usually large asymmetric organic cations and organic or inorganic anions) that are liquid at ambient temperatures [25]. In the scientific literature, ILs are frequently designated as environmentally friendly alternatives to toxic organic solvents due to their stability and low vapor pressure, leading to a generalization of their benign nature [26,27]. In addition, it is well documented that ILs synthesized from carefully selected, safe, naturally-derived materials can be environmentally benign with enhanced biodegradability and biocompatibility [28,29,30]. Such ILs can be based on low-cost and non-toxic cations, thus opening doors for their application in bioactive molecule extraction and even their further preservation [31,32]. Ionic liquid-based aqueous biphasic systems (IL-ABSs) represent liquid–liquid extraction techniques that allow for the recovery of target products from complex samples [33,34]. IL-ABSs can be applied in both the extraction and purification steps of high-value compounds from food waste. Moreover, through the wise selection of the phase-forming components and their compositions, IL-ABSs may be biocompatible, efficient, and low-cost [30,35]. Briefly, IL-ABSs consist of two aqueous-rich phases formed by mixing aqueous solutions of ionic liquid and a polymer or salt at appropriate concentrations [36]. Recent investigations have demonstrated that ILs with cholinium cations paired with appropriate anions present outstanding biodegradable and low-toxicity properties [30,37]. Moreover, some cholinium ILs possess a remarkable biomass dissolution ability and cellular disruption potential, which could be crucial assets in designing extraction platforms for polyphenol recovery [33]. Saha et al., in their 2022 comprehensive review, highlighted that a considerable number of ABSs have been explored for polyphenol extraction. However, these systems predominantly involve polymer–polymer, polymer–inorganic salt, or imidazolium-based ABSs [35]. In polymer-based ABSs, a limited difference in polarities between the two phases often hampers their widespread application in extraction processes. Ionic liquids, well-known for their tunability, can span the entire spectrum of hydrophilicity to hydrophobicity. Previous reports focused mainly on the use of imidazolium IL-ABSs have shown promise in extracting phenolic acids; however, imidazolium ILs are not ideal due to their lack of biocompatibility and potential toxicity [38]. There is still a scarcity of publications related to the investigation of cholinium-based ABSs for polyphenol extraction [39,40,41]. Ribeiro and coworkers documented their use of choline chloride-based ABSs for the extraction of polyphenols and saponins from plant extracts, achieving extraction efficiencies of around 30% [42]. In a separate study, Wang et al. employed cholinium ILs featuring anions derived from amino acids to extract flavonoids and pectin from ponkan peels at room temperature. They then utilized an ABS for the subsequent separation of these two substances [43]. Moreover, a study by Neves et al. (2019) explored the use of ABSs formed by cholinium-derived ILs and carbohydrates for the simultaneous separation of antioxidants and carbohydrates from food waste. The process demonstrated high extraction efficiencies for both carbohydrate (89–92%) and antioxidant (65–75%) activities from an expired vanilla pudding sample. The methodology allowed for the recovery of antioxidants and the recycling of ILs, representing a promising approach for food waste valorization [41].

Considering the aforementioned motivations, our goal was to create a novel integrated approach combining environmentally friendly alternative solvents, biocompatible ionic liquids, and aqueous biphasic systems for selective separation of key polyphenolic compounds from grape by-products. Specifically, we focused on trans-resveratrol (RSV), quercetin-3-O-glucoside (QC), and gallic acid (GA), which are prominent representatives of their respective classes and have garnered significant interest due to their anti-cancer, cardiovascular protective, antioxidant, and anti-free radical properties. We investigated the partition potential of IL-ABSs formed by cholinium-based ILs with different anions (dihydrogen phosphate [DHP], dihydrogen citrate [DHCit], chloride, lactate [Lac], vanillate [Van], gallate [Gal], and nicotinate [Nic]) in combination with polypropylene glycol 400 for target compounds. [Ch][DHP], [Ch][DHCit], and [Ch]Cl were chosen as excellent salting-out agents among cholinium salts, while [Ch][Lac], [Ch][Gal], [Ch][Van], and [Ch][Nic] were chosen to obtain more customizable ABSs by introducing different anions derived from natural acids. The phase diagrams of the liquid–liquid equilibria of each ABS were determined at room temperature, and the appropriate composition was set up for subsequent partition experiments. Screening of these ABSs was conducted to extract polyphenols, and the logarithmic distribution coefficients were calculated to provide insights into the extraction mechanism. To optimize operational parameters and establish an experimental setup for real samples, [Ch][DHP] was selected as the ABS component due to its strong salting-out ability and selectivity, with the anticipation that this choice would enhance phase separation and extraction efficiency. The influence of ABS compositions on the extraction performance of [Ch][DHP]-based ABSs was investigated. Subsequently, a carefully tailored ABS was employed to extract polyphenols from grape stem extract.

## 2. Materials and Methods

### 2.1. Materials

**Figure 2:**
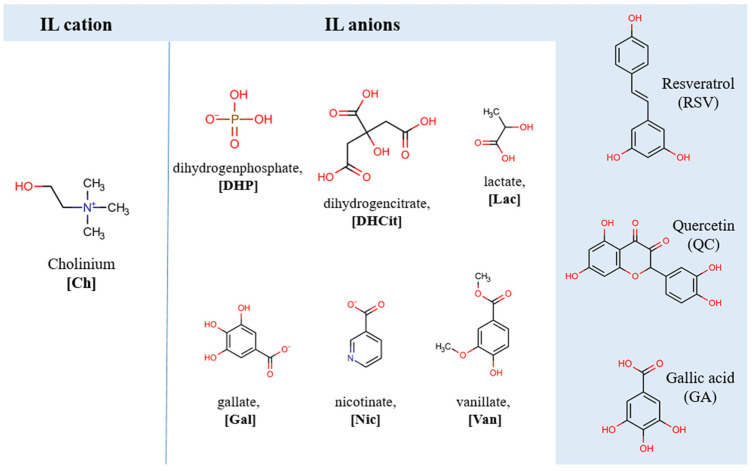
Chemical structures and abbreviations of the investigated cholinium ILs/salts and polyphenolic compounds.

Resveratrol (purity ≥ 99 wt%), quercetin (purity ≥ 99 wt%), and gallic acid (purity ≥ 99 wt%) were acquired from ExtraSynthese (Genay, France). Propylene glycol 400 (PPG400), choline hydroxide ([Ch][OH], purity ≥ 46 wt% in H_2_O), cholinium dihydrogen citrate ([Ch][DHCit]), HPLC-grade acetonitrile, and HPLC-grade ethanol were purchased from Sigma-Aldrich (St. Louis, MA, USA). Nicotinic acid (purity ≥ 99%) and vanillic acid (purity ≥ 98 wt%) were procured from ThermoFisher (Dreieich, Germany), and lactic acid (purity ≥ 90%) was acquired from Fluka Chemie (Buchs, Switzerland). Cholinium chloride ([Ch]Cl, purity ≥ 98%) was obtained from Across Organic (Geel, Belgium), while cholinium dihydrogen phosphate ([Ch][DHP], purity ≥ 99%) was provided by Iolitec (Heilbronn, Germany). Cholinium vanillate ([Ch][Van]), cholinium gallate ([Ch][Gal]), cholinium lactate ([Ch][Lac]), and cholinium nicotinate ([Ch][Nic]) were synthesized in this work by following well-established protocols, via neutralization of cholinium hydroxide with the corresponding acid—vanillic, gallic, lactic, and nicotinic acid, respectively [30,44]. The water content was initially removed using a rotary evaporator (R-210 Rotavapor System, BÜCHI Labortechnik AG, Flawil, Switzerland) for 4 h, followed by an additional drying process at 70 °C under vacuum conditions for 36 h. ILs were subjected to Karl Fischer titration with a Metrohm 831 Karl Fischer coulometer, Herisau, Switzerland, revealing a water content of less than 400 ppm. The chemical structures of the synthesized ILs were confirmed by Fourier transform infrared spectroscopy (Nicolet iS5 spectrometer fitted with an iD7 ATR Accessory, Thermo Fisher Scientific, Waltham, MA, USA). The obtained spectra, along with peak assignments, are provided in the Supplementary Materials (Figure S1a–d). Figure 2 represents the chemical structures and abbreviations of the studied cholinium ILs/salts, as well as the structures and abbreviations of the studied polyphenolic compounds.

### 2.2. Extraction of Polyphenolic Compounds

The capabilities of the investigated choline-based ABSs to extract RSV, QC, and GA were assessed. Considering previously established phase diagrams of ABSs, ternary mixtures within the biphasic region were prepared, containing 20 wt% salt/IL + 50 wt% PPG400 + 30 wt% of H_2_O +10 μL of polyphenol solution. The polyphenol solutions were prepared in 80 vol% ethanol, with concentrations of circa 2500 mg L^−1^ for each compound. After screening of the partition behaviors of the studied polyphenols for the ABSs under study, the most selective system was selected for further optimization studies. This involved investigating the influence of diverse tie-line lengths (TLLs) of 56.88, 76.09, and 101.43 and initial compositions of ABSs along the chosen tie line, i.e., different phase ratios of the extraction parameters of [Ch][DHP]/PPG-based ABSs. The selected compositions of ABSs and corresponding TLLs are given in the Supplementary Materials.

Each ABS was prepared in 1.5 mL microcentrifuge tubes by adding the appropriate amount of constituents to achieve a final weight of 1 g. All systems were vigorously agitated (Reax Top, Heidolph, Schwabach, Germany) and left for 2 h at 25 °C in a thermo-shaker incubator (ALEMADR-MSC, Colo Lab Experts, Novo Mesto, Slovenia). Following 5 min of centrifugation at 5000× *g* rpm (LLG-uniCFUGE 5, Meckenheim, Germany), the phases were carefully separated, and their weights and volumes were measured. After appropriate dilution and filtration using syringe filters (0.45 μm), the contents of polyphenols in the phases were quantified by high-performance liquid chromatography (HPLC), as described in Section 2.5.

The logarithmic distribution coefficients (logD) of the polyphenols were calculated as the ratio of the equilibrium concentration of each polyphenol in the PPG400-rich and IL/salt-rich phases, as follows:

(1) $$\mathrm{logD}=\log\;\frac{{\left[\mathrm{Polyphenol}\right]}_{\mathrm{PPG}400\text{-}\mathrm{phase}}}{{\left[\mathrm{Polyphenol}\right]}_{\mathrm{IL}/\mathrm{salt}\text{-}\mathrm{phase}}}.$$

Recovery efficiencies of resveratrol and quercetin (RE, %) toward the PPG400-rich phase were calculated as follows:

(2) $$\mathrm{RE}\;(\%)=\frac{{\left[\mathrm{Polyphenol}\right]}_{\mathrm{PPG}400\text{-}\mathrm{phase}}\cdot V_{\mathrm{PPG}400\text{-}\mathrm{phase}}}{{\left[\mathrm{Polyphenol}\right]}_{\mathrm{PPG}400\text{-}\mathrm{phase}}\cdot V_{\mathrm{PPG}400\text{-}\mathrm{phase}}+{\left[\mathrm{Polyphenol}\right]}_{\mathrm{IL}/\mathrm{salt}\text{-}\mathrm{phase}}\cdot V_{\mathrm{IL}/\mathrm{salt}\text{-}\mathrm{phase}}}\;\cdot 100$$

while the recovery efficiency of gallic acid (RE%) toward the IL/salt-rich phase was calculated using following equation:

(3) $$\mathrm{RE}\;(\%)=\frac{{\left[\mathrm{Polyphenol}\right]}_{\mathrm{IL}/\mathrm{salt}\text{-}\mathrm{phase}}\cdot V_{\mathrm{IL}/\mathrm{salt}\text{-}\mathrm{phase}}}{{\left[\mathrm{Polyphenol}\right]}_{\mathrm{PPG}400\text{-}\mathrm{phase}}\cdot V_{\mathrm{PPG}400\text{-}\mathrm{phase}}+{\left[\mathrm{Polyphenol}\right]}_{\mathrm{IL}/\mathrm{salt}\text{-}\mathrm{phase}}\cdot V_{\mathrm{IL}/\mathrm{salt}\text{-}\mathrm{phase}}}\;\cdot 100\;$$

where ${\left[\mathrm{Polyphenol}\right]}_{\mathrm{PPG}400\text{-}\mathrm{phase}}$ and ${\left[\mathrm{Polyphenol}\right]}_{\mathrm{IL}/\mathrm{salt}\text{-}\mathrm{phase}}$ represent the concentrations of RSV, QC, and GA, in the PPG400-rich and IL/salt-rich phases, respectively, and $V_{\mathrm{PPG}400\text{-}\mathrm{phase}}$ and $V_{\mathrm{IL}/\mathrm{salt}\text{-}\mathrm{phase}}$ are the volumes of the PPG400-rich and IL/salt-rich phases, respectively.

The selectivities (S) with respect to gallic acid were calculated according to the following equation:

(4) $$S_{\mathrm{GA}/\mathrm{Polyphenol}}=\frac{D_{\mathrm{GA}}}{D_{\mathrm{Polyphenol}}}$$

where the subscript “Polyphenol” stands for resveratrol or quercetin.

### 2.3. Recovery of Polyphenols from Grape Stems

Grapes collected from Quinta das Carvalhas, Pinhao (Regiao Demarcada do Douro, Portugal), were first separated into stems, seeds, and skins. All samples were freeze-dried, ground into powder, and stored in a desiccator. Two grams of the grape stem powder was mixed with 100 mL of a water:ethanol mixture (1:1, *v*:*v*), followed by stirring for 2 h and sonication for 5 min. The sample was centrifuged at 10,000× *g* rpm for 15 min, and the pellet was re-extracted. The supernatant was collected, and the solvent was evaporated under vacuum using a rotary evaporator at 40 °C. Using the HPLC analytical method, the concentrations of RSV and GA in grape stem extract were determined to be 5.2 and 8.9 μg g^−1^, respectively, while quercetin was not detected (Figure S6 in Supplementary Materials). The resulting grape stem extract (5 mg) was added to the ternary ABS mixture with the composition indicated in the monophasic region of the phase diagram. This mixture was stirred continuously for at least two hours at a controlled temperature of 25 °C. Subsequently, an aqueous solution of [Ch][DHP] was introduced to the mixture to achieve a biphasic system composition of 40 wt% PPG400 and 25 wt% [Ch][DHP]. The solution was thoroughly mixed and allowed to equilibrate at room temperature for 2 h, followed by centrifugation at 5000× *g* rpm for 10 min and physical phase separation ([Ch][DHP]-rich and PPG400-rich phases). The phases were diluted, filtered through syringe filters (0.45 µm), and subjected to HPLC analysis. The experimental stages are summarized in Table 1.

### 2.4. Solubility Determination

The solubilities of RSV in a PPG400/water system with volume ratios of 80:20 and the solubility of GA in a [Ch][DHP]/water system with volume ratios of 50:50 were determined. Initially, RSV and GA were added to corresponding solutions in excess while continuously mixing at a constant temperature of 25 °C for 12 h. After centrifugation at 6000 rpm, the saturated solution was sampled, diluted, filtered, and quantified by UV spectroscopy using an LLG-uniSPEC2 spectrophotometer at wavelengths of 280 nm for GA and 307 nm for RSV. Quantification was carried out using calibration curves (R^2^ > 0.99 in all cases) for each analyzed compound within the concentration range of 2.5 to 50 mg L^−1^.

### 2.5. Quantification of Polyphenols

The quantification of polyphenols was carried out using HPLC (Knauer, Berlin, Germany), which includes a DAD detector and an Ascentis C18 (SUPELCO), Darmstadt, Germany, column with a particle size of 5 µm and dimensions 250 mm × 4.6 mm. Chromatographic separation of the target polyphenols occurred at a constant flow rate of 1 mL min^−1^, the injection volume for all samples was 20 μL, and the temperature of the column was fixed at 45 °C, with 10% acetonitrile (*v*/*v*) as mobile phase A and pure acetonitrile as mobile phase B. For partition studies, isocratic conditions were used for the determination of each compound (A:B = 60:40). Quantification in a complex sample after extraction from the real sample was achieved using a gradient mode, varying the mobile phase from 100% A to 100% B for 30 min. The DAD detector was set to measure at 307 nm for RSV, 250 nm for QC, and 280 nm for GA, with retention times of 14.60, 12.50, and 2.60, respectively.

## 3. Results and Discussion

### 3.1. Liquid–Liquid Equilibria of Cholinium-Based ABSs

Seven extensively researched cholinium-based aqueous biphasic systems were selected to assess their efficacy in extracting polyphenols of varying polarities from aqueous solutions. These systems comprised three cholinium salts ([Ch][DHP], [Ch][DHCit], and [Ch]Cl) and four cholinium ionic liquids ([Ch][Lac], [Ch][Nic], [Ch][Van], and [Ch][Gal]), each combined with PPG400 and water to form biphasic systems for polyphenol extraction. Following the conventional definition of ionic liquids, cholinium-based compounds are categorized as such when their melting point is below 100 °C, as exemplified by [Ch][Gal], [Ch][Van], [Ch][Lac], and [Ch][Nic]. Conversely, cholinium salts like [Ch][DHP], [Ch][DHCit], and [Ch]Cl exhibit melting points above 100 °C [45]. Initial experiments involved precisely determining the mixture compositions capable of forming two phases suitable for extraction studies.

**Figure 3:**
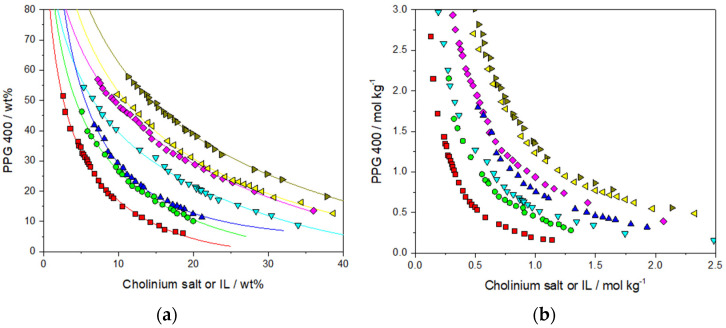
Ternary phase diagrams composed of cholinium salt or IL + PPG400 + H_2_O at 25 °C. The compositions are given in (**a**) weight percents and (**b**) molality units (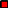, [Ch][DHP]; 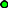, [Ch][Lac]; 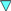, [Ch][DHCit]; 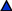, [Ch]Cl; 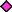, [Ch][Gal]; 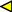, [Ch][Nic]; 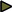, [Ch][Van]).

While there exists literature on liquid–liquid equilibria for selected cholinium-based ABSs with PPG400, there is a lack of comparative data regarding the ability of the studied series of ILs to form ABSs [30,37,46,47,48]. Therefore, we opted to investigate and compare solubility data to mitigate potential discrepancies stemming from temperature variations and variations in ionic liquid purities. In every ABS studied, the cholinium salt/IL and water predominantly form the lower phase, while the upper phase mainly consists of polypropylene glycol 400 (PPG400) and water. The respective phase diagrams are illustrated in Figure 3a, with the experimental data on weight fractions obtained via the cloud point method available in Table S1 of the Supplementary Materials. To further analyze the structural effects of the IL, the phase diagrams are also represented in molality units, as shown in Figure 3b, to account for the differences in molecular weights of the ILs. Regression parameters (A, B, and C) were calculated using the Merchuk equation [49], which is detailed as Equation (S1) in the Supplementary Materials, with values and their standard deviations listed in Table S2, indicating a high level of correlation. These parameters were applied to a [Ch][DHP]-based ABS to derive various tie-line (TL) data through a gravimetric method, as outlined in the Supplementary Materials. Figure S3 showcases a series of tie lines for this system, defined for different initial compositions and corresponding to increasing total tie-line lengths (56.88, 76.09, and 101.43). Additional information on these tie lines is available in Table S2 of the Supplementary Materials. An increase in hydrophobicity within the upper phase is observed, as highlighted by the rise in IL concentration at higher TLL values. For the tie line with a TLL of 101.43, two more ABSs were identified, each with different phase ratio volumes. These data on specific tie lines in the [Ch][DHP]-based ABS are crucial for the optimization studies presented in Section 3.3, providing insights into the system’s performance under various conditions. In systems with larger biphasic regions, the ability of cholinium salts or ILs to undergo liquid–liquid separation is enhanced. Given that the cholinium cations remain constant across all salts/ILs, differences in phase behavior are mainly due to the distinct properties of the anions. This is in contrast to inorganic salt-based ABSs, where demixing is primarily driven by the salting-out effect of ions with high charge density. The demixing mechanism in IL polymer ABSs is recognized as more complex, stemming from the interaction dynamics among the three components, namely polymer, IL, and water [45,50]. There is a competitive interaction for water molecules between PPG400 and the IL ions, as well as among the ions themselves, with the IL anions more prone to forming hydration complexes compared to the IL cations [51].

According to the phase diagrams depicted in Figure 3b, the ability of cholinium salts/ILs to form ABSs decreases as follows: [Ch][DHP] > [Ch][Lac] > [Ch][DHCit] > [Ch]Cl > [Ch][Gal] [Ch][Nic] > [Ch][Van]. The ABS trend of high-melting-point cholinium salt is in accordance with data reported in existing literature [30,37]. [Ch][DHP] demonstrated the most prominent ability to expel PPG400. Earlier studies have highlighted that the primary distinction between cholinium salts and ILs with higher and lower melting points, which correlates with their capability to form ABSs and their affinity for water, lies in the values of their anion polar surfaces or logK_ow_ values [45]. Anion polar surfaces represent the total surface area of all polar atoms, mainly oxygen and nitrogen, along with their bonded hydrogens. Typically, salts with higher melting points exhibit greater polar surface values due to a more localized charge. Although [DHCit]^−^ has the highest polar surface charge among the studied cholinium compounds (132.13 Å^2^), it is not a stronger salting-out agent than [Ch][DHP] (77.76 Å^2^). As explained in the work of Pereira et al., [DHCit]^−^ anions exhibit intramolecular hydrogen bonds between the hydroxyl hydrogen atoms and one of the oxygens of the central carboxyl group, which decreases their interaction with water and, consequently, the respective salting-out ability [32,45].

In the context of ILs with lower melting temperatures, the ability to form ABSs declines in the following order: [Ch][Lac] > [Ch][Gal] > [Ch][Nic] > [Ch][Van]. [Ch][Lac] demonstrates the strongest capacity among cholinium ILs to induce ABS formation, while [Ch][Van] has the lowest. This sequence does not align perfectly with the logK_ow_ values of the ILs’ anions, which are −3.74, −2.48, −3.03, and 1.50 for [Lac]^−^, [Gal]^−^, [Nic]^−^, and [Van]^−^, respectively [52]. The case of vanillate correlates well with its logK_ow_ value, being the most hydrophobic and, thus, the least effective in ABS formation. However, despite nicotinate being more hydrophilic than gallate, it shows a lower tendency to form ABSs. Additionally, the polar surface areas of these anions (57.53, 97.99, 50.19, and 55.76 Å^2^ for [Lac], [Gal], [Nic], and [Van], respectively) also do not mirror the decreasing ABS formation trend, as gallate has a higher value than lactate, while vanillate surpasses nicotinate. Nevertheless, [Gal] lags behind [Lac] in forming ABSs, and [Van] is weaker than [Nic]. This indicates that the formation of ABSs in these low-melting-point ILs with PPG is determined by a complex interplay of interactions with the polymer and water [30,53]. The gallate anion possesses four hydroxyl groups on its ring, providing four hydrogen donors and five hydrogen bond acceptors in its molecule. This contributes to its high polar surface area, which is even greater than that of the [DHP] anion, known as the strongest salting-out agent. The fact that [Ch][Gal] is a considerably weaker ABS phase former than [Ch][DHP] suggests that the solvation of IL anions may be largely affected by intermolecular interactions in ionic liquids, i.e., ion-pair binding energies. A multitude of ab initio studies have established that the binding energies between the cation and anion of an IL are closely related to the IL’s melting point and its transport properties, like conductivity and viscosity [54,55,56,57]. Tot et al. showed that these binding energies play a significant role in determining the structure-making properties of ILs, i.e., their interactions with water [58]. Contrary to common expectations, they found that functionalization of an IL with a hydroxyl group does not enhance its structure-making properties, a key factor in the formation of ABSs, where the ability to influence structure is crucial. This could explain the lower ability of [Ch][Gal] to form ABS in comparison to [Ch][Lac]. A good indication of higher binding energy and more robust molecular packing of [Ch][Gal] is its higher melting temperature ([Ch][Gal] is solid, while [Ch][Lac] is liquid at room temperature; Figure S2). Additionally, even though [Ch][Nic] has a high logK_ow_ value, it acts as a weak salting-out agent. The presence of π electrons in [Ch][Nic], capable of forming strong hydrogen bond interactions with the ether and oxygen atoms of PPG, might significantly contribute to its reduced capability in facilitating the formation of ABSs [30].

### 3.2. Extraction of Polyphenolic Compounds

**Figure 4:**
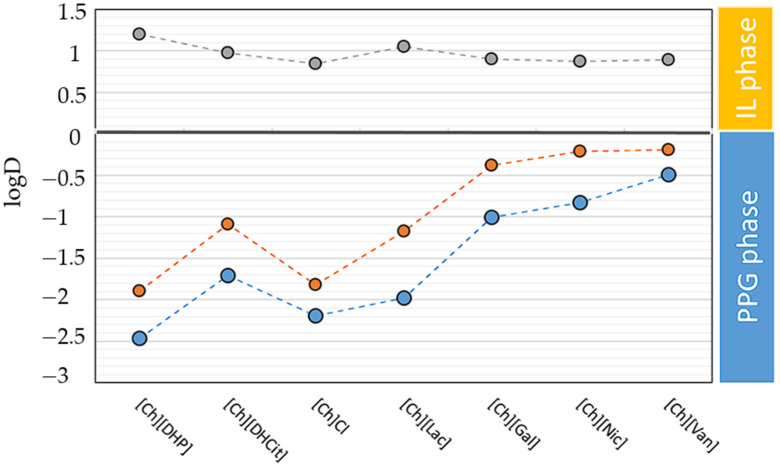
Distribution coefficients of polyphenols in cholinium-based ABSs at 25 °C and atmospheric pressure (RSV, blue circles; QC, orange circles; GA, gray circles).

In selecting appropriate ILs to evaluate the recovery performance of selected polyphenols in this work, several factors were considered. Having in mind our goal, a strong focus was placed on selecting highly biocompatible ILs that are soluble in water. This led to the choice of ILs based on the quaternary ammonium cation, specifically the cholinium cation. Additionally, the selection of anions was also crucial in tailoring the physicochemical properties and interaction capabilities of the ILs to achieve adequate solvation ability for target natural compounds. Thus, the focus was also on anions derived from natural sources, such as from plant natural acids (gallic acid, vanillic acid, lactic acid, and nicotinic acid). Moreover, ILs with anions similar to those of the target compounds, such as gallate, vanillate, or nicotinate, allow for enhanced recovery selectivity [30]. We also considered more common commercial cholinium salts, such as [Ch][DHP], [Ch]Cl, and [Ch][DHCit], since they all have excellent salting-out ability among cholinium ILs. Therefore, by strategically considering both cations and anions, we aimed to select highly environmentally benign and biocompatible ABS constituents that possess desirable characteristics for our intended applications. To evaluate the effects of distinct ILs on the partitions of polyphenols in the proposed IL-based ABSs, the following appropriate composition of ternary ABS mixtures was selected: 20 wt% IL + 50 wt% PPG400 + 30 wt% water. This composition of the ternary mixtures was selected based on the previously determined phase diagrams with the aim of encompassing the biphasic range of each ABS, which is defined by the system with the lowest ability to undergo liquid−liquid demixing, namely the [Ch][Van]-based ABS. Moreover, when choosing the mixture compositions of the studied extraction ABSs, we endeavored to obtain similar phase ratios (V_IL_phase_/V_salt_phase_~0.5) of the systems to facilitate comparison. We evaluated two key parameters, namely the logarithmic distribution coefficients (logD) and recovery efficiency (RE), both crucial for discussing the performance of each ABS in extracting specific polyphenols. LogD values serve as an effective tool to delve into the partitioning mechanisms of polyphenols, revealing the complex interplay of interactions among ABS components―PPG400, the IL, the solute, and water. Meanwhile, recovery efficiency enabled us to identify the most efficient and selective systems, guiding the design of integrated processes for recovery of polyphenols from food by-products. The data presented as logarithmic distribution coefficients (logD values) in Figure 4 clearly demonstrate the varying affinities of the selected polyphenols for different phases in ABSs. LogD values greater than 0 signify a tendency of the polyphenol to favor the PPG-rich phase, whereas values less than 0 denote a predisposition towards the hydrophilic IL-rich phase.

The overall pattern in the partitioning of the analyzed polyphenols reveals that RSV and QC tend to migrate to the PPG-rich phase (‒0.49 < logD_RSV_ < ‒2.47 and ‒0.19 < logD_QC_ < ‒1.89, respectively), while GA exhibits a preference for the IL-rich phase (0.84 < logD_GA_ < 1.2). Among studied cholinium-based ABSs, the partitioning behavior of QC and RSV is aligned with the following sequence of ILs/salts: [Ch][DHP] > [Ch]Cl > [Ch][Lac] > [Ch][DHCit] > [Ch][Gal] > [Ch][Nic] > [Ch][Van]. This order is in close agreement with the ABS formation aptitude, with the exception of [Ch][DHCit]. At first glance, these results suggest that the distributions of hydrophobic polyphenols in cholinium-based ABSs are determined by the hydrophobic properties of the phases and the salting-out effect of cholinium salts/ILs (Figure S5). However, an exception is noted in systems using cholinium-based salts with melting temperatures above 100 °C. Specifically, in the [Ch][DHCit] ABS, the distribution coefficients are lower compared to those in the [Ch][DHP] and [Ch]Cl-based ABS, contradicting their expected behavior based on the phase diagram and the hydrophobicity of the PPG-rich phase. This odd behavior of [Ch][DHCit] might stem from specific intramolecular interactions within the [DHCit]^−^ anions, which reduce its salting-out effect. A similar phenomenon was observed in a study by Dimitrijević et al., where [Ch][DHCit]/Pluronic PE6200 showed the lowest distribution coefficients for hydrophobic alkaloids like caffeine, theophylline, and theobromine [32]. The authors attributed this to the IL and water content in the Pluronic-rich phase. Considering PPG is a more hydrophobic polymer than Pluronic PE6200, it is plausible that the reduced salting-out capability of the [DHCit] anion is less marked than in Pluronic systems. This explains why logD values of [Ch][DHCit]/PPG ABSs are not lower in comparison to ABSs with lower-melting-point cholinium ILs. Additionally, the pronounced tendency of RSV in comparison to QC to migrate dominantly towards the upper PPG phase in each ABS is noticeable if comparing their determined distribution coefficients for each ABS. According to octanol–water partition coefficient values, RSV is the most lipophilic among the studied compounds (logK_ow_ = 3.40 and logK_ow_ = 2.70 for RSV and QC, respectively), which explains its high values of distribution coefficients (Table S4).

Contrary to stronger salting-out agents, the molecular mechanisms underlying the polyphenol partitions in [Ch][Lac], [Ch][Gal], [Ch][Nic], and [Ch][Van] ABSs are more complex, and specific interactions between components must be considered [32,44]. Therefore, the distribution pattern in these ABS is not only dominated by the hydrophobicity and salting out. Instead, it is largely a direct consequence of the favorable (or non-favorable) interactions that occur between the polymer, ILs, and polyphenols [20]. RSV and QC are more distributed toward the PPG-rich phase but with lower values of K than in the high-melting-point choline salt- based ABSs. Diminished K values in [Ch][Gal], [Ch][Nic], and [Ch][Van] ABSs can be attributed to the lower relative difference of hydrophobicity between the PPG and IL phases and π-π stacking interactions between IL anions (phenolic moieties) and aromatic structures of solutes.

Contrary to other compounds, GA predominantly partitions into the more hydrophilic IL-rich phases, as indicated by its negative octanol–water partition coefficients (logK_ow_ values ranging from 0.65 to −2.48 across pH 3.2–7.2). Furthermore, within the operational pH range of the ABSs, the fraction of negatively charged GA molecules reaches as high as 98.5% (Figure S4), emphasizing that the charged form of GA is prevalent, highlighting the significance of electrostatic interactions in its migration to the IL-rich phase. As shown in Figure 4, the logD values of GA do not completely mirror the hydrophobicity trend of ABSs outlined by their binodal curves. LogD values of GA align with the hydrophilicity of the IL-rich phase in cholinium salt ABSs in the following order: [Ch][DHP] > [Ch][DHCit] > [Ch]Cl. Intriguingly, [DHCit]-IL does not exhibit the same behavior as seen with non-charged, hydrophobic polyphenols, aligning with results from other studies [32,45]. In room-temperature IL systems, the distribution trends of GA align with their respective phase diagrams, showing a pattern of [Ch][Lac] > [Ch][Gal] > [Ch][Nic] > [Ch][Van]. However, the logD values for GA in these systems are not markedly lower compared to those in cholinium salt ABSs, even though their IL-rich phases are relatively less hydrophilic. This suggests that GA’s distribution is influenced by a more intricate set of mechanisms, involving various interactions such as hydrogen bonding, π-π stacking, electrostatic forces, and van der Waals interactions. Notably, [Gal], [Nic], and [Van] anions establish additional π-π interactions with the aromatic part of GA that could compensate for their lower hydrophilicity.

**Figure 5:**
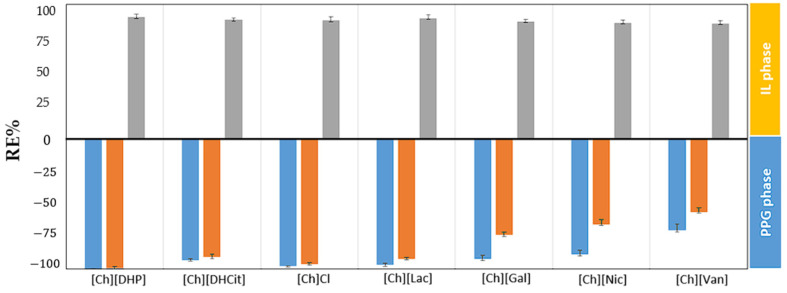
Recovery efficiencies (RE%) of GA (gray bar), RSV (blue bar), and QC (orange bar) in the studied ABSs at 25 °C.

The obtained recovery efficiencies (RE%) in the IL-rich phases for gallic acid and PPG-rich phases for quercetin and resveratrol are depicted in Figure 5 (detailed results are provided in the Supplementary Materials). The RE% values align more closely with the ability to form an ABS, as opposed to the trends observed in logD values, following the following sequence: [Ch] [DHP] > [Ch][Lac] > [Ch][DHCit] > [Ch]Cl > [Ch][Gal] > [Ch] [Nic] > [Ch][Van]. The highest REs for each polyphenol were obtained for the [Ch][DHP]-based ABS, namely 99.71 ± 0.60% for RSV and 98.92 ± 1.02% for QC toward the PPG-rich phase and 92.31 ± 2.31% for GA toward the IL-rich phase. Slightly lower values (reduced by less than 5%) were obtained for [Ch][Lac], [Ch][DHCit], and [Ch]Cl. Lower recovery efficiencies for QC and RSV were found with ILs containing anions from natural polyphenol acids, namely [Ch][Gal], [Ch][Nic], and [Ch][Van], ranging from 70.29 ± 4.89% to 92.27 ± 3.07% for RSV and from 56.04 ± 3.12% to 73.65 ± 3.07% for QC. This decrease in REs is due to the less hydrophobic nature of the PPG phases in these ABSs and the capacity of the anionic ILs to engage in π–electron interactions and hydrogen bonding with RSV and QC, which could be a significant factor in their reduced recovery efficiency for hydrophobic molecules. On the other hand, RE values of GA for different ABSs are generally steadier and higher, which is in accordance with their high distribution coefficients.

**Figure 6:**
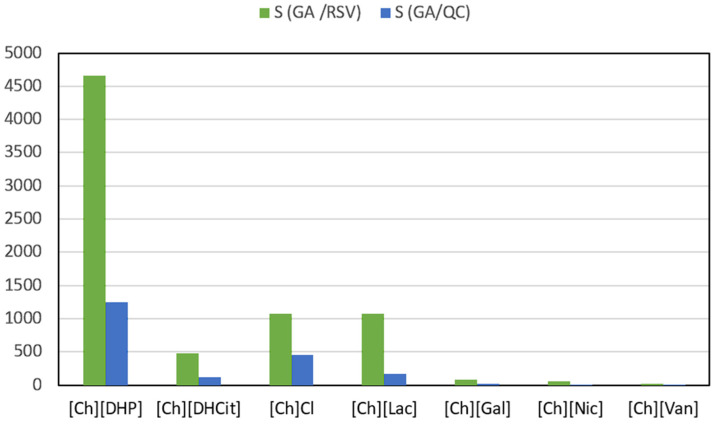
Selectivity parameters in studied ABSs at 25 °C.

The ABS comprising [Ch][DHP]/PPG400 enables the highest partition coefficients for QC and RSV towards the PPG-rich phase and simultaneously yields the lowest coefficients for GA. This suggests that an effective separation of the examined polyphenols can be realized by selecting appropriate ABS components. Figure 6 illustrates the selectivity of the systems under study in separating resveratrol or quercetin from gallic acid. Among the evaluated ABSs, the [Ch][DHP]-based system stands out as the most selective in separating gallic acid from the other polyphenols, with a an SGA/RSV selectivity ratio exceeding 4500 and S_GA/QC_ surpassing 1000.

Investigations of cholinium IL-based systems for the extraction and separation of polyphenols have been reported in the literature. For example, Xavier [39] employed ABSs based on [Ch]Cl and different surfactants to separate ferulic acid and p-coumaric acid from rice husk hydrolysate. Both compounds used in their study migrated to the surfactant-rich phase (with yields over 90%), which differs from the results we obtained for GA, which partitions dominantly in the IL-rich phase. Wang et al. [43] explored ABSs composed of cholinium-based ILs for the extraction of flavonoids and pectin from ponkan peel, achieving selective separation, with flavonoids favoring the IL-rich phase and pectin remaining in the salt-rich phase. While this study successfully separated polyphenols from polysaccharides, it did not accomplish selective separation among different polyphenols. Similarly, Neves et al. utilized cholinium-based ABSs for extraction and separation of antioxidants and carbohydrates from food waste [41]. The process demonstrated high efficiencies in separating antioxidants (65–75%) from an expired vanilla pudding sample. Several works have studied the extraction and purification of polyphenols using ABSs based on imidazolium ILs [33]. In these systems, polyphenols partitioned preferably to the IL-rich phase, so low selectivities were achieved. Additionally, imidazolium ILs are not considered ideal for polyphenol extraction due to concerns regarding their biocompatibility and potential toxicity. Generally, variations in the type of ionic liquids, polyphenols, samples, and experimental designs across different studies make direct comparisons of the obtained results with literature data challenging.

### 3.3. Recovery of Polyphenols from Grape Stems and Designing an Integrated Extraction and Separation Process

Our partition studies showed that cholinium IL/PPG ABSs hold considerable promise for the one-step extraction and separation of various polyphenols, including RSV, QC, and GA. By carefully choosing the anions in the IL’s chemical structure, we were able to delve into the extraction mechanisms, enhance ABS performance, and fine-tune selectivity. To further evaluate the capability of cholinium-based ABSs for the direct extraction of polyphenols from grape stem extract, it was imperative to carefully choose the extraction system and fine-tune the operational parameters, notably the TLL and the composition mixture within the same tie line. The selection process for the optimal cholinium-based ABS as a polyphenol extraction/separation platform involved evaluating the extraction efficiency and selectivity of the systems. Among the evaluated cholinium salts, cholinium dihydrogen phosphate emerged as the ideal choice. The [Ch][DHP]-based ABS showcased exceptional performance in achieving the highest extraction efficiency and selectivity for each target compound in a single-step process. The salting-out effect, which is important in the formation of an ABS and the distribution of polyphenols, is a defining feature of [Ch][DHP]. By choosing an appropriate ABS composition, it is possible to establish considerably long tie lines. These longer tie lines contribute to improved recovery efficiencies and concentration factors while also reducing cross-contamination between the phases. This is achieved by ensuring that the PPG-rich phase contains minimal [Ch][DHP] and that the salt-rich phase is predominantly composed of water and salt.

**Figure 7:**
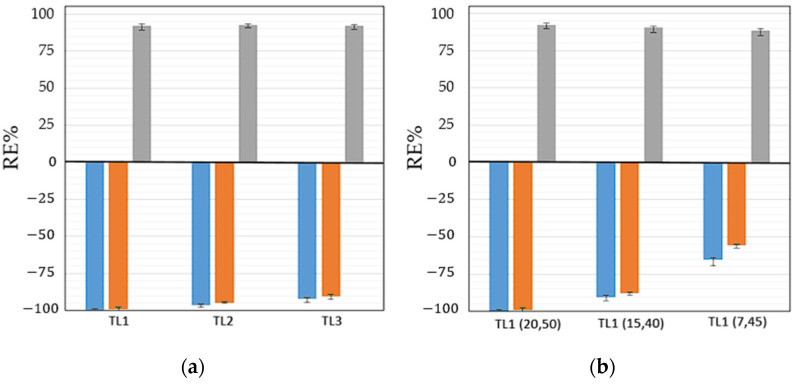
Impact of different TLLs (**a**) and different initial compositions along the same TL (**b**) on the extraction efficiencies of RSV (blue bar), QC (orange bar), and GA (gray bar) in the [Ch][DHP] + PPG400 + H_2_O ABS at 25 °C.

The influence of the TLL on polyphenol partitions was assessed by varying the concentrations of ABS constituents as presented in Figure S3. Figure 7a depicts how the increase in TLL impacts RSV, QC, and GA extraction. By increasing the TLL under the tested conditions (polyphenol content = 2.5 g per L of ABS, pH~6, and T = 25 °C) increases of circa 7%, 9%, and 2% in RE_RSV_, RE_QC_, and RE_GA_ were obtained, respectively. The influence of the TLL on RE_RSV_ and RE_QC_ is primarily owing to the salting-out capability of [Ch][DHP] that also rises with the increase in the TLL. Adjusting the initial compositions of ABSs within the same tie line results in varying volume ratios of the coexisting phases, while their individual compositions remain unchanged. This variation affects the REs, as they are influenced by the phase volume ratios. Specifically, systems with different volume ratios of the PPG phase relative to the [Ch][DHP] phase, such as 0.50, 0.40, and 0.20, were examined. These systems, all with tie lines approximately 101 in length, are illustrated in Figure 7b. The findings show a decline in REs as the phase volume ratios decrease (for example, REs dropped from 99.71 ± 0.60% to 65.20 ± 3.89% for RSV, from 98.92 ± 1.02% to 55.70 ± 2.13% for QC, and from 92.31 ± 2.31% to 88.7 ± 3.91% for GA). Therefore, optimizing the volume ratios of the aqueous phases by reducing the volume of the PPG400 phase and minimizing the use of [Ch][DHP], without sacrificing extraction efficiency, is crucial.

**Figure 8:**
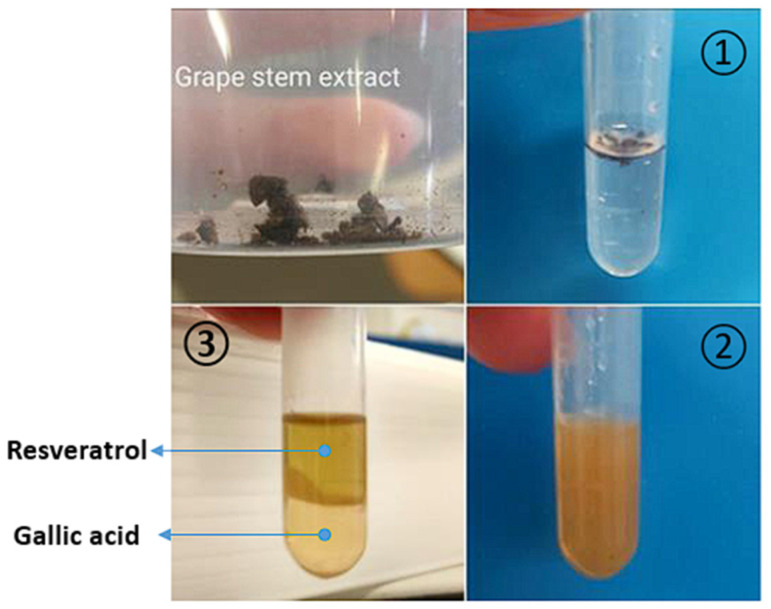
Extraction process from grape stem extract: 1—adding extract to IL + PPG400 aqueous solution; 2—mixing; 3—separation of the phases.

**Figure 9:**
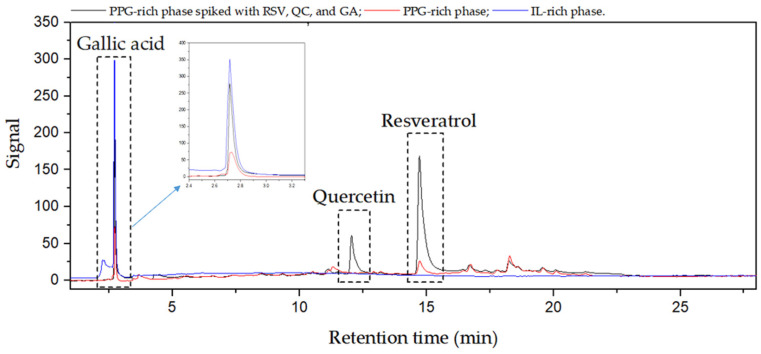
Chromatograms of IL-rich (blue line) and PPG-rich (red line) phases after solid–liquid extraction and liquid–liquid separation from grape stem using a [Ch][DHP]/PPG400 ABS. The black line represents a PPG phase spiked with a standard solution of RSV, QC, and GA.

Guided by the results from the optimization studies, polyphenols were extracted from grape stem extract with specific conditions in mind based on previously determined extraction parameters. These included the use of a [Ch][DHP] + PPG400 ABS, a temperature of 25 °C, a pH of approximately 6, a tie-line length of around 101, and grape stem extract quantities of about 5 mg per g of ABS, with the volume ratio of the salt phase relative to the PPG phase being approximately 0.4. In the process, mixtures of PPG, [Ch][DHP], and water in the monophasic region were directly employed in the extraction of valuable compounds from grape by-products, then stirred. Subsequently, an aqueous solution of [Ch][DHP] was added to the mix of PPG400, water, and grape stem extract to achieve an ABS composition of 40 wt% PPG400 and 25 wt% [Ch][DHP] at room temperature, as depicted in Figure 8. After stirring and equilibration, the ABS mixtures were centrifuged. Remarkably, RSV was completely extracted from the grape stem extract into the PPG-rich phase, achieving an exceptional 100% recovery efficiency. The RE for GA towards the [Ch][DHP] phase was 78.64%, and quercetin was not detected in the grape stem extract. Figure 9 shows the chromatograms of both ABS phases after extraction of the selected polyphenols using the [Ch][DHP]/PPG400 ABS, showing that RSV is dominantly presented in PPG-rich phase, while GA migrates toward the IL-rich phase. The application of optimized operational parameters for the extraction of polyphenols from grape stems resulted in an enhanced extraction of RSV, achieving a higher recovery efficiency than that observed during the optimization process. Conversely, the recovery efficiency for GA is lower than that found in our partition experiments. This could be due to the reduced hydrophilicity of the IL-rich phase, possibly because of the presence of additional polar compounds in the extract that compete with [Ch][DHP] for water molecules.

RSV is a highly hydrophobic compound and, thus, presents a low solubility in pure water (39 mg/L [59]). Considering that the upper PPG phase of the ABS mainly consists of PPG and water (with less than 0.1 wt% [Ch][DHP]), we aimed to determine the solubility of RSV in a PPG/water system matching the composition of the PPG phase with the purpose of estimating the capacity of the [Ch][DHP]/PPG ABS for RSV extraction. We achieved a solubility of 100 g/L for RSV, although this was not its maximum solubility. The notably high solubility of RSV in the PPG-rich phase suggests the feasibility of using this system to recover substantial amounts of hydrophobic compounds or for application in continuous processes before reaching saturation. Given that RSV content in grape stems ranges from 4.6 to 15.4 mg/kg [12] and based on the determined solubility of RSV in the PPG phase, it would theoretically be possible to process a minimum of 6.4 to 21.7 kg of plant material until RSV saturation if 1 L of the PPG rich phase is reached. Similarly, a high capacity for GA recovery is also expected. The maximal solubility of GA in the [Ch][DHP]-rich phase (around 50 wt% [Ch][DHP]) was found to be 139.8 g/L, which is significantly higher than its solubility in water (14.38 g/L). Claudio et al. corroborated the exceptional ability of ILs to act as hydrotropes through the formation of ionic liquid–biomolecule aggregates [60]. This characteristic is crucial from the perspective of developing new strategies for polyphenol recovery. Specifically, GA from approximately 12 kg of material could potentially be recovered in 1 L of the [Ch][DHP] phase, showcasing the efficacy of this method.

**Figure 10:**
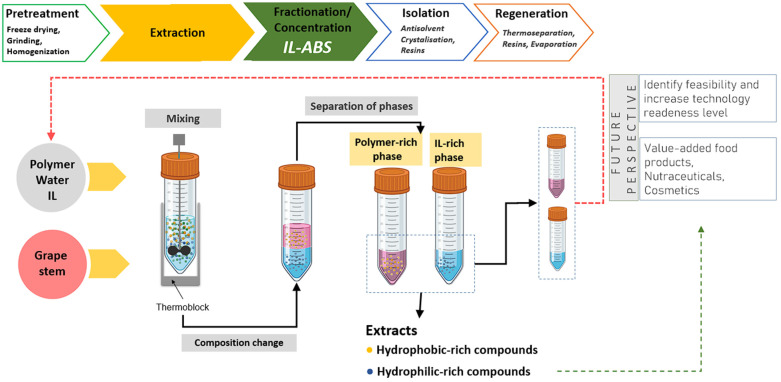
Schematic representation of the proposed integrated platform to extract and recover value-added compounds from grape by-products using ABSs composed of cholinium-based ILs and PPG400.

The evaluated ABS is able to remarkably recover, concentrate, and separate value-added compounds directly from grape stem samples and largely contributes to the green concept of the designed integrated separation platform. All components applied as ABS phase formers in the proposed integrated concept are benign from a toxicological point of view. PPG400 is a harmless and highly biocompatible polymer approved as a food additive by the FDA. Cholinium-based salts and ILs are safe, generally non-toxic, and biodegradable ionic compounds. A conceptual design of the developed integrated platform to extract and recover value-added compounds from food by-products is schematically shown in Figure 10.

After the extraction and separation step, bioactive polyphenols need to be isolated from the PPG and IL matrix. Although the isolation step is outside of the scope of the experimental study reported here, some suggestions are provided with respect to how to isolate and reuse the ABS components (dashed lines in Figure 10) to further emphasize the sustainable character of the process. RSV could be easily recovered from the PPG-rich phase by antisolvent crystallization, whereby adding water as antisolvent can significantly lower the solubility of hydrophobic polyphenol [61]. Isolation from the IL matrix could be achieved using affinity resins. For example, Rosa et al. achieved pigment isolation from the IL and PPG matrix by a solid-phase extraction approach using affinity resins, with high recoveries of over 96% for betalains and 98% for chlorophylls [34]. Only by addressing and guaranteeing the recycling of phase-forming components will it be possible to be economically competitive with other well-established techniques.

## 4. Conclusions

In this work, we proposed an integrated approach based on ABSs to simultaneously extract, separate, and concentrate selected polyphenols (resveratrol, quercetin, and gallic acid) from grape stem extract. For that purpose, phase diagrams composed of PPG400 and seven different salting-out agents ([Ch][DHP], [Ch]Cl, [Ch][DHCit], [Ch][Lac], [Ch][Gal], [Ch][Nic], and [Ch][Van]) were determined at 25 °C and under atmospheric pressure. We established the aptitude of salts to generate ABS with PPG400 in the following order: [Ch][DHP] > [Ch]Cl > [Ch][Lac] > [Ch][DHCit] > [Ch][Gal] > [Ch][Nic] > [Ch][Van]. While the aptitude of higher-melting-point cholinium salts for ABSs follows the well-established ability of their anions to be solvated by water and act as salting-out species, lower-melting-point cholinium ILs completely follow neither logK_ow_ nor polar anion surface parameters, as described earlier in the literature. As discussed, IL ion-pair binding energies play an important role in determining the interactions of IL anions with water and with the polymer, which are crucial in ABS formation. Next, extraction experiments were performed to determine the partition behavior of each polyphenol in the investigated ABSs, and different distribution patterns were observed for the investigated polyphenols. [Ch][DHP] exhibited the highest extraction efficiencies of above 95% for RSV and QC toward the PPG phase and 90% for GA in the IL phase. Also, this system stands out as the most selective in separating GA from the other polyphenols, with a S_GA/RSV_ selectivity ratio exceeding 4500 and S_GA/QC_ surpassing 1000. The distribution patterns in other ABSs are not solely governed by hydrophobicity and salting-out effects but are also a result of the specific interaction between the polymer, ILs, and polyphenols. After screening the extraction ability of each ABS, key operational conditions, specifically the TLL and mixture composition along the same tie line, were established to optimize extraction performance from a real sample. It was shown that RSV was completely extracted from the grape stem extract into the PPG-rich phase, achieving an exceptional 100% recovery efficiency. The RE for GA towards the [Ch][DHP] phase was 78.64%, and quercetin was not detected in the grape stem extract. The lower recovery of GA could be due to the reduced hydrophilicity of the IL-rich phase. The evaluated ABS is able to remarkably recover, concentrate, and separate value-added compounds directly from grape stem samples and largely contributes to the green concept of the designed integrated separation platform.

## Figures and Tables

**Table 1:** Experimental stages and appropriate system compositions (wt%).

Experimental Stage			IL	Polymer	Polyphenol Sample	T (°C)	System Composition		
							IL		PPG400
**IL** **Synthesis**		1	[Ch][Val]	/	/	25		/	
		2	[Ch][Gal]						
		3	[Ch][Lac]						
		4	[Ch][Nic]						
**Determination of ABS** **diagrams**		1	[Ch][DHP]	PPG400	/	25		/	
		2	[Ch][DHCit]						
		3	[Ch]Cl						
		4	[Ch][Lac]						
		5	[Ch][Gal]						
		6	[Ch][Nic]						
		7	[Ch][Van]						
**Partition studies**		1	[Ch][DHP]	PPG400	Standard ~2500 mg per L	25	~20		~50
		2	[Ch][DHCit]						
		3	[Ch]Cl						
		4	[Ch][Lac]						
		5	[Ch][Gal]						
		6	[Ch][Nic]						
		7	[Ch][Van]						
**Optimization** **studies**	TL	1	[Ch][DHP]	PPG400	Standard ~2500 mg per L	25	~20		~50
		2					~16		~40
		3					~12		~30
	ABS composition (phase ratio)	1	[Ch][DHP]	PPG400	Standard ~2500 mg per L	25	~20		~50
		2					~25		~40
		3					~40		~6
**Recovery from grape stems**		1	[Ch][DHP]	PPG400	5 mg stem extract per mL	25	~25		~40
**Solubility**		1	[Ch][DHP]	/	GA standard	25	~50		/
		2	/	PPG400	RSV standard	25	/		~80

## Data Availability

The original contributions presented in the study are included in the article/Supplementary Material, further inquiries can be directed to the corresponding author.

## Supplementary Materials

The following supporting information can be downloaded at: https://www.mdpi.com/article/10.3390/foods13060954/s1, Figure S1a–d: FTIR spectra of the synthesized ionic liquids; Figure S2: Synthesized [Ch][Gal] (left) and [Ch][Lac] (right); Figure S3: Ternary phase diagram composed of [Ch][DHP] + PPG 400 + H2O at 25°C with illustrated TLs; Figure S4: Speciation diagram of gallic acid; Figure S5: Chromatograms of investigated polyphenolic in [Ch][DHP] or [Ch][Nic] -rich phase (black line) and PPG400-rich phase (red line) at 280nm; Table S1: Experimental binodal mass fraction data for the salt/IL (X) + PPG400 (Y) + H_2_O ABS at 25°C and at p = 0.1 MPa.; Table S2: Correlation parameters, standard deviations and determination coefficients (R2) of the salt/IL (X) + PPG400 (Y) + H_2_O ABS obtained by the Merchuk equation at 25°C and at p = 0.1 MPa); Table S3: Experimental tie-lines data in percentage weight fraction for the ABS composed of ([Ch][DHP] + PPG400 (Y) + H_2_O (Z)) at 25°C, pH = 6, and 0.1 MPa and volume ratios (Vr).; Table S4: The properties of the polyphenolic compounds. Figure S6: Chromatograms of grape stem extract (red line) and spiked grape stem extract (black line) in ethanol at 280 nm. References [49,62] are cited in the supplementary materials.
