# Supplementary material for: Green Extraction Strategy Using Bio-Based Aqueous Biphasic Systems for Polyphenol Valorization from Grape By-Product
Source: Foods. 2024 Mar 21;13(6):954. doi: 10.3390/foods13060954 (PMC10969810; doi:10.3390/foods13060954)
Supplement: Supplementary file 1 [file foods-13-00954-s001.zip › foods-2916001-supplementary.pdf]

Supporting Information

Green Extraction Strategy Using Bio-Based Aqueous Biphasic Systems for Polyphenol Valorization from Grape By-Product

Aleksandra Dimitrijević [1,*], Slađana Marić [1], Ana Jocić [1], Danijela Tekić [1], Jasmina Mušović [1] and Joana S. Amaral [2,3]

[1] Vinča Institute of Nuclear Sciences—National Institute of the Republic of Serbia, University of Belgrade, Mike Petrovica Alasa 12–14, 11000 Belgrade, Serbia; sladjana.maric@vin.bg.ac.rs (S.M.); ana.jocic@vin.bg.ac.rs (A.J.); danijela.tekic@vin.bg.ac.rs (D.T.); jasmina.musovic@vin.bg.ac.rs (J.M.)
[2] Centro de Investigação de Montanha (CIMO), Instituto Politécnico de Bragança, Campus de Santa Apolónia, 5300-253 Bragança, Portugal; jamaral@ipb.pt
[3] Laboratório Associado para a Sustentabilidade e Tecnologia em Regiões de Montanha (SusTEC), Instituto Politécnico de Bragança, Campus de Santa Apolónia, 5300-253 Bragança, Portugal

***Corresponding author:** Aleksandra Dimitrijević – daleksandra@vin.bg.ac.rs

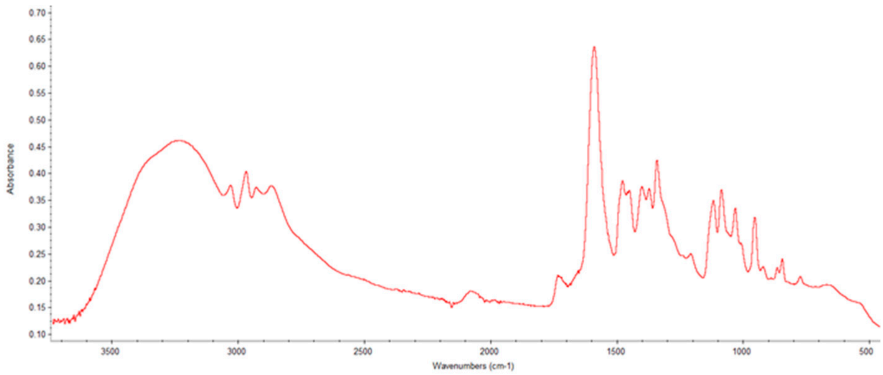

Figure S1a. FTIR spectra of the synthesized [Ch][Lac].

IR (neat): 3246 (OH); 2968; 1589 (COO); 1477 (CH_3); 1375 (CH_3); 1087 (C-CH_3); 954 (C-C-O).

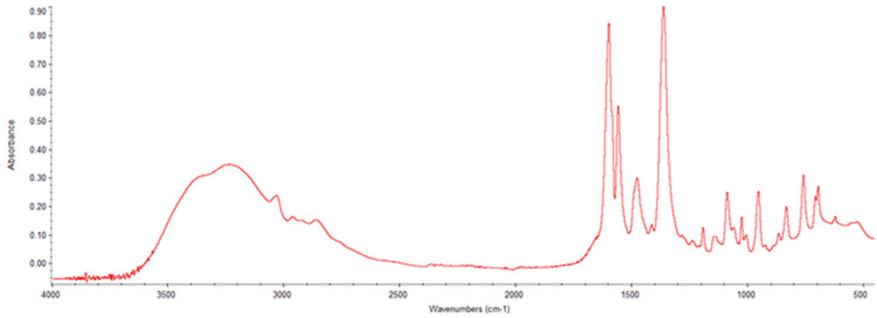

Figure S1b. FTIR spectra of the synthesized [Ch][Nic].

IR (neat): 3232 (OH); 1598 (C=C); 1559 (NH); 1478 (CH_3); 1362 (COO); 1192 (stretching C-OH, from Ph-OH); 1088 (C-C-N); 1025; 953 (C-C-O); 832 (C-C); 758, 695.

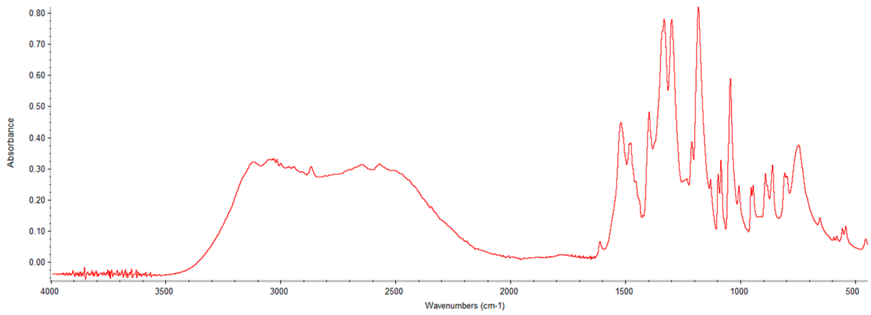

Figure S1c. FTIR spectra of the synthesized [Ch][Gal].

IR (neat): 3038 (OH); 2572, 1522 (COO); 1332 (COO^-); 1184 (CN); 1044; 945; 861; 758.

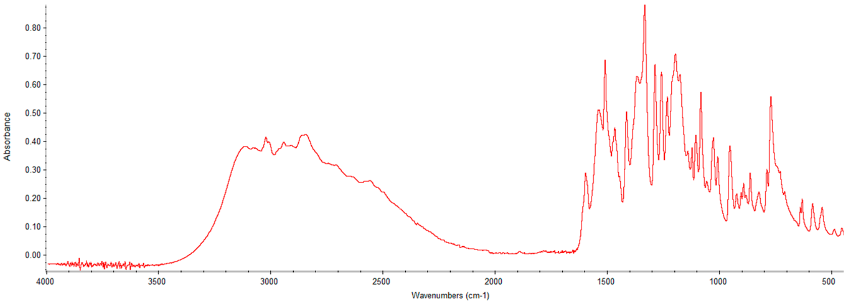

Figure S1d. FTIR spectra of the synthesized [Ch][Van].

IR (neat): 3022 (OH); 2845; 1534 (COO); 1333 (COO); 1196 (CN); 1088; 1083; 1027; 771; 632.

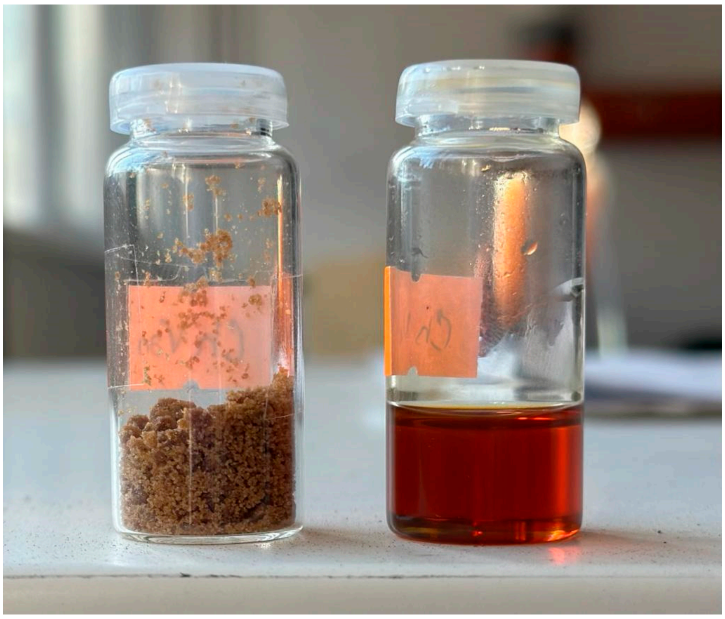

Figure S2. Synthesized [Ch][Gal] (left) and [Ch][Lac] (right).

Determination of the phase diagrams of ABS

The phase diagrams of ABS composed of each salt/IL, PPG400 and water were determined by applying the cloud point titration method at (25±1) °C and atmospheric pressure (0.1 MPa) following established protocol [1]. The obtained experimental solubility data from the biphasic phase diagrams were fitted using the empirical Merchuk equation:

$$Y=A \exp\left[\left(BX^{0.5}\right)-\left(CX^{3}\right)\right] \qquad (S1)$$

where Y and X are, respectively, the PPG400 and IL/salt weight fractions percentages; and A, B, and C are regression parameters determined using the OriginPro 8.5 software.

Tie lines (TLs) were determined by the lever–arm rule according to Merchuk et al. [2] The following system of four equations (Egs. S2-S5) with four unknown values was solved applying the MathCad 15.0 software:

$$Y_{PPG400} = A\ \exp\left[\left(BX_{PPG400}^{0.5}\right) - \left(CX_{PPG400}^{3}\right)\right] \quad (S2)$$

$$Y_{IL} = A\ \exp\left[\left(BX_{IL}^{0.5}\right)\text{-}\left(CX_{IL}^{3}\right)\right] \quad (S3)$$

$$Y_{PPG400} = \left(\frac{Y_M}{\alpha}\right) - \left(\frac{(1\text{-}\alpha)}{\alpha}\right)Y_{IL} \quad (S4)$$

$$X_{PPG400} = \left(\frac{X_M}{\alpha}\right) - \left(\frac{(1\text{-}\alpha)}{\alpha}\right)X_{IL} \quad (S5)$$

where subscripts PPG400, IL, and M correspond to the PPG400-rich phase, the IL-rich phase and the mixture, respectively; α is the ratio between the mass of the top phase and the total mass of the mixture. The system solution results in the composition (wt%) of the IL and PPG400 in the top and bottom phases. Each tie-line length (TLL) was calculated using the equation:

$$TLL = \sqrt{(X_{PPG400} - X_{IL})^2 + (Y_{PPG400} - Y_{IL})^2} \quad (S6)$$

pH measurements. pH values of ABS were measured using Orion-Star A111 pH Benchtop Meter Kit at 25±1 ºC and within ± 0.01 pH units. The calibration of the pH meter was carried out with three standard buffers (pH values of 10.10, 7.00 and 4.01).

Table S1. Experimental binodal mass fraction data for the salt/IL (X) + PPG400 (Y) + H_2O ABS at 25°C and at p = 0.1 MPa.

[Ch][DHP]		[Ch][DHCit]		[Ch]Cl	
100·X	100·Y	100·X	100·Y	100·X	100·Y
2.50	51.67	5.32	54.32	6.73	41.81
2.84	46.29	6.49	50.83	7.29	40.47
3.49	40.78	7.40	47.45	8.08	37.32
4.52	36.51	7.81	45.21	8.55	32.98
4.66	35.08	8.81	42.69	8.94	31.56
4.90	34.62	9.64	40.45	9.84	29.34
5.08	32.60	12.79	33.69	10.59	27.67
5.25	32.26	14.94	31.05	11.11	25.43
5.46	31.30	16.11	28.18	11.70	24.47
5.74	30.50	16.61	26.71	12.22	23.16
5.81	29.82	17.85	24.69	12.97	21.85

6.01	29.01	18.35	23.33	13.31	21.27
6.18	28.05	19.34	22.36	15.57	17.85
6.70	25.87	20.25	21.44	16.51	16.71
7.26	23.56	20.37	20.87	17.19	15.56
7.65	21.82	20.77	20.52	17.78	15.07
8.28	20.35	21.00	20.21	18.76	14.11
8.56	19.30	21.58	19.35	18.25	14.44
9.01	18.49	23.32	17.07	19.91	12.42
9.25	17.71	22.24	18.33	21.10	11.29
10.41	15.03	25.90	15.38		
12.21	12.55	28.31	13.38		
13.03	11.42	30.38	12.00		
14.15	9.85	33.94	8.73		
15.34	8.69	42.24	5.88		
16.11	7.46				
17.52	6.60				
18.52	6.29				

[Ch][Lac]		[Ch][Gal]		[Ch][Nic]		[Ch][Van]	
100·X	100·Y	100·X	100·Y	100·X	100·Y	100·X	100·Y
5.08	46.29	7.23	56.85	9.84	51.97	11.28	57.83
5.82	39.83	7.56	55.68	10.69	50.12	11.92	55.88
6.32	38.12	7.81	53.99	12.00	47.58	12.39	54.63
7.06	35.60	8.31	52.41	12.73	45.47	13.03	52.99
8.14	32.11	9.05	50.83	13.93	42.74	13.70	51.92
9.64	27.98	9.31	50.08	14.47	41.56	13.96	50.88
10.29	26.18	9.64	49.34	16.52	36.56	14.17	49.47
10.05	26.49	10.26	47.58	17.32	35.21	15.03	49.05
10.46	25.54	10.56	46.92	18.30	33.09	15.20	47.63
11.29	23.22	11.12	45.90	19.66	31.25	15.92	46.32
11.79	21.73	11.53	45.25	21.11	29.02	16.25	45.36
12.55	20.78	12.26	43.72	21.92	27.51	16.81	44.87
13.12	19.84	12.73	42.54	22.98	25.93	17.41	43.56
13.87	18.87	13.29	40.96	24.22	24.78	17.79	42.79
14.70	18.24	14.08	39.32	25.23	23.60	18.77	40.69
14.87	16.65	14.38	37.27	26.00	23.11	18.97	40.09
15.83	15.57	15.37	35.43	26.73	22.29	19.74	39.18
16.94	14.22	16.35	33.63	27.44	21.80	20.43	38.09
17.32	13.66	17.23	32.45	28.25	20.89	21.75	35.60
17.78	12.63	18.09	31.42	29.11	19.91	22.00	35.14
18.67	12.42	18.86	30.22	38.63	12.64	22.77	33.85
19.93	10.09	20.17	28.76	34.38	16.33	23.71	32.00
19.17	11.26	21.37	27.23	30.76	17.96	28.08	27.18
		22.72	25.18			29.82	25.65
		23.92	24.09			31.82	23.82
		25.19	22.78			37.77	18.22
		28.08	19.91			42.71	15.29
		35.99	13.51				

[1]The standard uncertainty of the measured percentage weight fraction is $u(100 \cdot y) = u(100 \cdot x) = 0.066$, $u(T) = 1$ °C. Relative standard uncertainty: $u_r (p) = 1.5\%$

Table S2. Correlation parameters, standard deviations and determination coefficients (R^2) of the salt/IL (X) + PPG400 (Y) + H_2O ABS obtained by the Merchuk equation at 25°C and at p = 0.1 MPa)

IL	A ± σ	B ± σ	C ± σ	R^2
[Ch][DHP]	145.34 ± 4.88	-0.66 ± 0.02	$(7.20 \pm 1.44)\cdot 10^{-5}$	0.9962
[Ch][DHCit]	135.66 ± 4.03	-0.39 ± 0.02	$(1.16 \pm 0.16)\cdot 10^{-5}$	0.9971
[Ch]Cl	243.28± 19.74	-0.67 ± 0.03	$(-8.10 \pm 8.22)\cdot 10^{-5}$	0.9955
[Ch][Lac]	155.94 ± 7.61	-0.55 ± 0.02	$(-2.89 \pm 0.72)\cdot 10^{-5}$	0.9966
[Ch][Gal]	145.97 ± 4.02	-0.34 ± 0.01	$(6.88 \pm 1.26)\cdot 10^{-6}$	0.9971
[Ch][Nic]	180.72 ± 7.33	-0.38 ± 0.01	$(4.43 \pm 1.00)\cdot 10^{-6}$	0.9976
[Ch][Van]	190.05 ± 4.77	-0.35 ± 0.01	$(3.22 \pm 0.46)\cdot 10^{-6}$	0.9987

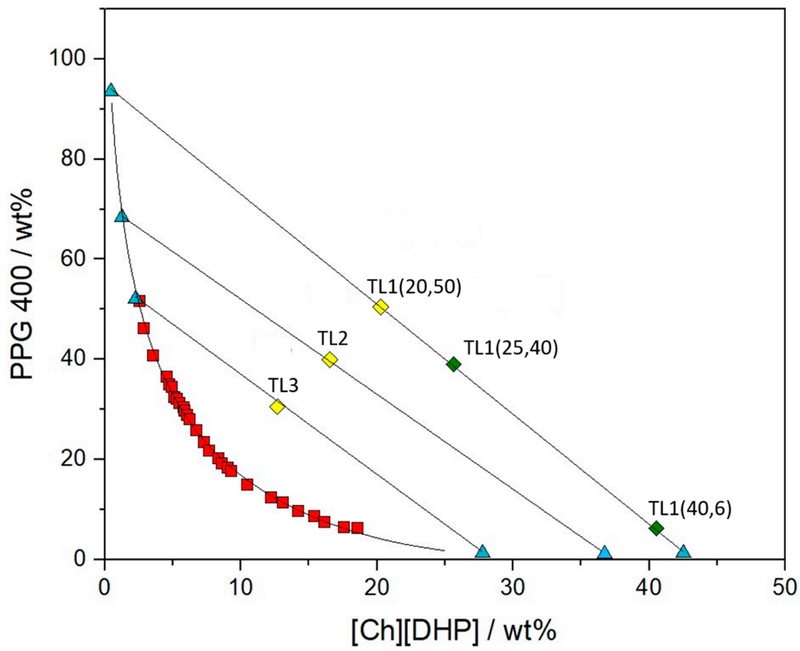

Figure S3. Ternary phase diagram composed of [Ch][DHP] + PPG 400 + H_2O at 25°C with illustrated TLs; ■, binodal curve data; ▲, TL data; ◆ and ◆, initial ABS compositions.

Table S3. Experimental tie-lines data in percentage weight fraction for the ABS composed of ([Ch][DHP] + PPG400 (Y) + H_2O (Z)) at 25°C, pH ~ 6, and 0.1 MPa and volume ratios (Vr).

[Ch][DHP]/PPG400	ABS composition		PPG-rich phases			IL-rich phases			TLL	Vr
	100·X	100·Y	100·X	100·Y	100·Z	100·X	100·Y	100·Z		
TL1(20,50)	20.25	50.44	0.44	93.57	5.99	42.51	1.27	56.22	101.43	0.5
TL1(25,40)	25.63	39.01	0.44	93.57	5.99	42.51	1.27	56.22	101.43	0.4
TL1(40,6)	40.52	6.19	0.44	93.57	5.99	42.51	1.27	56.22	101.43	0.2
TL2	16.54	39.93	1.25	68.41	30.34	36.72	1.09	62.19	76.09	0.5
TL3	12.68	30.59	2.26	52.11	45.63	27.76	1.27	71.97	56.88	0.4

Table S4. The properties of the polyphenolic compounds.

**Compound**	**Chemical structure**	[1]**pK_{a1}/pK_{a2}**	[1]**logK_{ow}** (pH=7)	[1]**HBA**	[1]**HBD**
Resveratrol	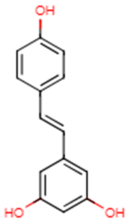	8.49	3.40	3	3
Quercetin	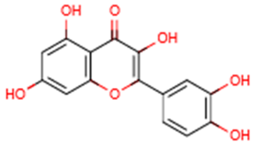	7.58	2.70	7	4
Gallic acid	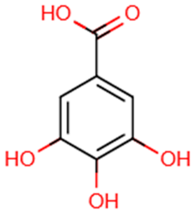	3.94	-2.21	5	4

[1]from Chemicalize- online cheminformatics platform by ChemAxon's, https://chemicalize.com/app/calculation

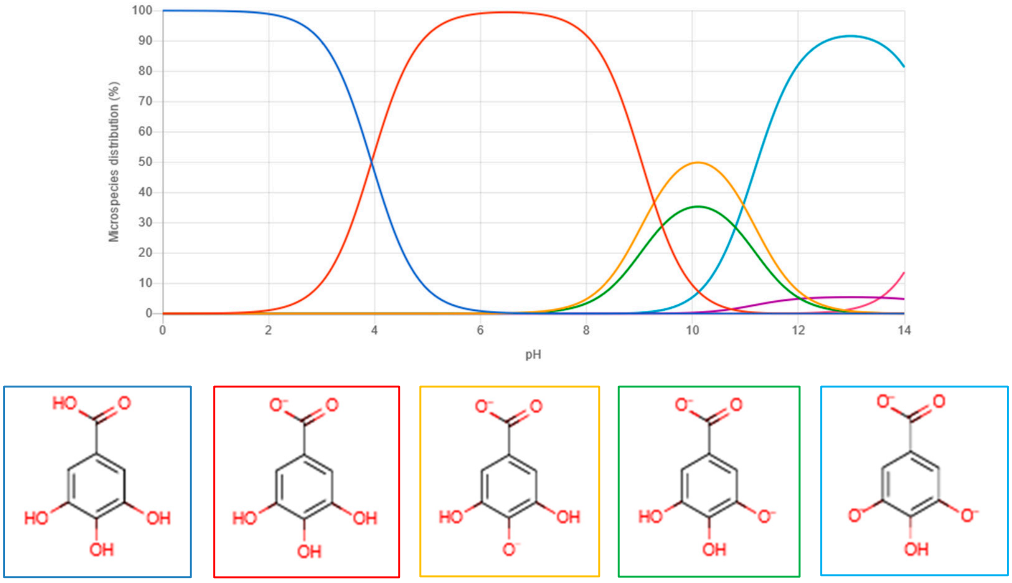

Figure S4. Speciation diagrams of gallic acid (from Chemicalize- online cheminformatics platform by ChemAxon's, https://chemicalize.com/app/calculation).

a) RESVERATROL

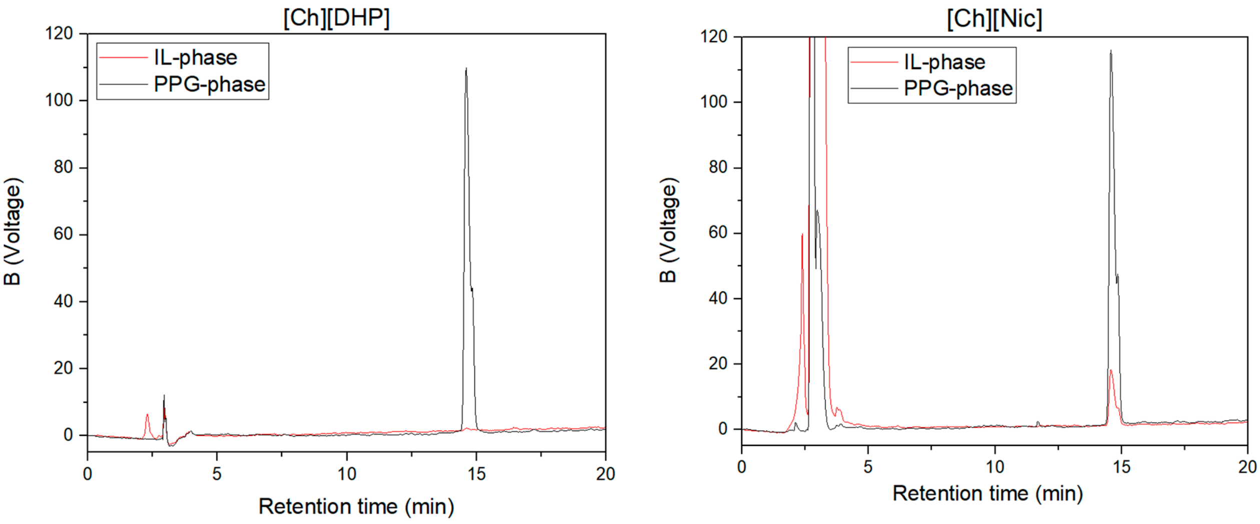

b) QUERCETIN

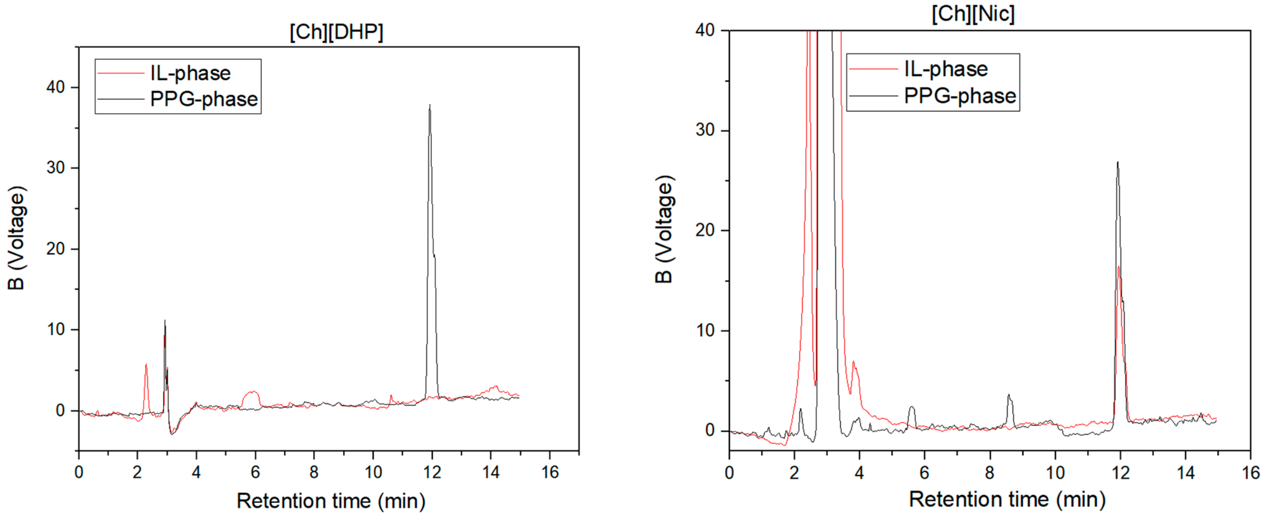

Figure S5. Chromatograms of investigated polyphenolic in [Ch][DHP] or [Ch][Nic] -rich phase (black line) and PPG400-rich phase (red line) at 280nm.

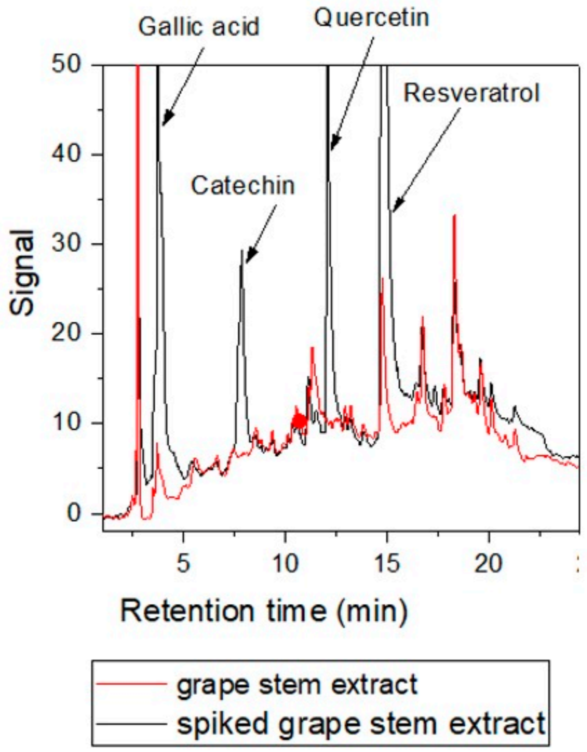

Figure S6. Chromatograms of grape stem extract (red line) and spiked grape stem extract (black line) in ethanol at 280nm.

[1] K.E. Gutowski, G.A. Broker, H.D. Willauer, J.G. Huddleston, R.P. Swatloski, J.D. Holbrey, R.D. Rogers, Controlling the aqueous miscibility of ionic liquids: Aqueous biphasic systems of water-miscible ionic liquids and water-structuring salts for recycle, metathesis, and separations, J. Am. Chem. Soc., 125 (22) (2003) 6632.

[2] J.C. Merchuk, B.A. Andrews, J.A. Asenjo, Aqueous two-phase systems for protein separation, J. Chromatogr. B Biomed. Sci. Appl., 711 (1–2) (1998) 285.
